# Drug development advances in human genetics‐based targets

**DOI:** 10.1002/mco2.481

**Published:** 2024-02-09

**Authors:** Xiaoxia Zhang, Wenjun Yu, Yan Li, Aiping Wang, Haiqiang Cao, Yuanlei Fu

**Affiliations:** ^1^ School of Pharmacy, Key Laboratory of Molecular Pharmacology and Drug Evaluation (Yantai University), Ministry of Education, Collaborative Innovation Center of Advanced Drug Delivery System and Biotech Drugs in Universities of Shandong Yantai University Yantai Shandong China; ^2^ Yantai Key Laboratory of Nanomedicine & Advanced Preparations, Yantai Institute of Materia Medica Yantai Shandong China; ^3^ Shandong Laboratory of Yantai Drug Discovery, Bohai Rim Advanced Research Institute for Drug Discovery Yantai Shandong China; ^4^ State Key Laboratory of Drug Research & Center of Pharmaceutics, Shanghai Institute of Materia Medica, Chinese Academy of Sciences Shanghai China

**Keywords:** drug development, drug target, genetic variation, genome‐wide association studies, whole‐exome sequencing, whole‐genome sequencing

## Abstract

Drug development is a long and costly process, with a high degree of uncertainty from the identification of a drug target to its market launch. Targeted drugs supported by human genetic evidence are expected to enter phase II/III clinical trials or be approved for marketing more quickly, speeding up the drug development process. Currently, genetic data and technologies such as genome‐wide association studies (GWAS), whole‐exome sequencing (WES), and whole‐genome sequencing (WGS) have identified and validated many potential molecular targets associated with diseases. This review describes the structure, molecular biology, and drug development of human genetics‐based validated beneficial loss‐of‐function (LOF) mutation targets (target mutations that reduce disease incidence) over the past decade. The feasibility of eight beneficial LOF mutation targets (PCSK9, ANGPTL3, ASGR1, HSD17B13, KHK, CIDEB, GPR75, and INHBE) as targets for drug discovery is mainly emphasized, and their research prospects and challenges are discussed. In conclusion, we expect that this review will inspire more researchers to use human genetics and genomics to support the discovery of novel therapeutic drugs and the direction of clinical development, which will contribute to the development of new drug discovery and drug repurposing.

## Graphical abstract

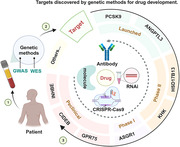

## 1 INTRODUCTION

Most drugs work with “target” molecules in the body to modulate the biological function. Drug targets are the direct binding sites of drugs and biomolecules in the body, including receptors, enzymes, ion channels, transporters, nucleic acids, and other biomolecules.^1^ Drug development is a long and expensive process, with a high degree of uncertainty from drug target identification to clinical trials and access to the market. A major challenge to drug development is the high failure rate in clinical development, which increases the cost and slows down the development of new drugs.^2^ In the drug development stage, the effect of a drug depends largely on the choice of target. The key to modern new drug development lies in finding and identifying drug targets, which has become the focus of intense competition in innovative drug development.^1^ Therefore, the discovery of new targets with high clinical translation rates is a prominent support for innovative breakthroughs in drug development.

Many diseases are genetically linked. Genetic research can not only lead to a better understanding of disease‐gene associations, but also to the identification of potential drug targets that can lead to the development of new treatments. Generally speaking, drug targets with human genetic evidence are more likely to be clinically translatable^3^ and thus enter phase II/III clinical trials or be approved for marketing more quickly,^4^, ^5^, ^6^, ^7^ which will likely significantly reduce the cost of drug development and drive the rapid development of the pharmaceutical industry. Therefore, it is critical to understand the biological background (especially the mechanism of action) of the key genes that contribute to the disease before commencing on a high‐throughput drug discovery screen.^8^

Since the publishing of the first human genome sequence, advances in high‐throughput sequencing and information technology has indeed brought about major breakthroughs in the study of disease pathogenesis.^7^, ^9^ In addition to a deeper understanding of genetic mutations in cancers and rare diseases,^10^, ^11^, ^12^ the association between diseases and potential therapeutic molecular targets has been deepened through the analysis of multiple loci associated with different complex diseases and important physiological traits.^13^, ^14^ This brings new opportunities for new target discovery methods and drug discovery and development, in particular, the development of therapeutic drugs in a more targeted manner to improve therapeutic efficacy and lay the groundwork for individualized treatment of diseases.^9^, ^15^ Currently, genetic data and technologies such as genome‐wide association studies (GWAS), whole‐exome sequencing (WES), and whole‐genome sequencing (WGS) have identified and validated many potential molecular targets related to diseases.^16^, ^17^ The association between loss‐of‐function (LOF) mutations and disease has also been the subject of several studies.^18^, ^19^, ^20^, ^21^, ^22^ LOF mutation is a mutation in a gene in the genome that causes the protein it encodes to lose its normal function.^18^ For most LOF mutations lead to an increased risk of disease, such as *KLF13* LOF mutations leading to familial congenital heart disease.^19^, ^20^, ^23^ Fortunately, some LOF mutations result in a reduced risk of disease. These benign mutations can produce a natural disease‐protective effect, providing crucial support for the development of targeted drugs. By mimicking these rare LOF variants, we can explore new therapeutic strategies and develop targeted drugs for specific diseases. A well‐known example is that LOF mutations in PCSK9 reduce serum low‐density lipoprotein (LDL) cholesterol levels and prevent coronary heart disease,^21^ which greatly contributed to the development of PCSK9 inhibitors, with two approved by the United States Food and Drug Association by 2015.^24^, ^25^

With this in mind, there is a growing trend among researchers and developers to integrate drug development with human genetics approaches. Its main objective is to utilize insights from human genetics research to accelerate the development of new drugs. In this review, we first summarize some of the common genetic techniques available for target discovery and drug development. Second, the structure, molecular biology, and drug development of human genetics‐based validated beneficial LOF mutation targets (target mutations that reduce the incidence of disease) over the last decade have described in detail. The review highlights the feasibility of eight beneficial LOF mutation targets (PCSK9, ANGPTL3, ASGR1, HSD17B13, KHK, CIDEB, GPR75, and INHBE) as targets for drug discovery and discusses their research prospects and challenges. This review does not go into too much detail on deleterious variants, as they have been reviewed elsewhere in the context of cancer and rare diseases.^9^, ^11^, ^26^, ^27^, ^28^, ^29^, ^30^, ^31^ In conclusion, we expect that more researchers will be inspired by these targets. We also reckon more people to use human genetics and genomics to support the discovery of novel therapeutic agents and the direction of clinical development, thus facilitating the development of new drug discovery and drug repurposing.^32^

## 2 HUMAN GENETICS METHODOLOGIES USED IN DRUG DEVELOPMENT

The discovery and characterization of drug targets are extreme challenging. There is no perfect method for finding targets quickly and accurately. But with the relentless efforts of scientists, enormous methods have been generated that can be used for drug target discovery and characterization. Traditional drug discovery is medicinal chemistry‐ and pharmacology‐driven and consists primarily of screening and validation based on cellular and animal models.^33^ However, most of these approaches are time‐consuming and not always effective.^33^ Drug target discovery usually relies on advanced technological tools and research methods. In recent years, with the rapid development of biotechnology and computer technology, new target discovery methods and techniques have emerged, bringing new opportunities and challenges to drug discovery.^33^

For example, bioinformatics‐based target prediction methods focus on finding potential drug targets by analyzing the sequence and structure of proteins^34^; proteomics‐based target prediction methods focus on finding potential targets by studying the structure and interaction network of proteins^35^; genomics‐based target exploration methods are mainly used to discover disease‐related genes or mutations through the study of the human genome, to further exploration of targets interacting with them. This approach is based on data such as gene expression profiling and genome sequencing to discover potential targets by comparing the differences between diseased and normal tissues.^1^ The main genetic methods currently used for drug development are GWAS, WES, and WGS.^36^, ^37^, ^38^, ^39^

### 2.1 Genome‐wide association studies (GWAS)

With the rapidly declining cost of high‐throughput genotyping, GWAS has been widely used to search for genetic risk factors that predispose individuals to complex diseases or traits.^40^ GWAS is a multicenter, large‐sample, iterative validation approach to study the association of genes with complex diseases at the human genome‐wide level using single‐nucleotide polymorphisms (SNPs) as molecular genetic markers.^32^, ^41^, ^42^

GWAS aims to identify common genetic variants associated with traits and diseases.^43^ The development of GWAS has transformed the field of genetics of complex diseases and deepened the understanding of heritable traits.^44^ GWAS improves the diagnosis and treatment of undiagnosed complex diseases such as primary immunodeficiencies.^45^ Several loci associated with different complex diseases (e.g., hypercholesterolemia, type II diabetes mellitus, coronary artery disease [CAD], obesity, nonalcoholic fatty liver disease [NAFLD]/nonalcoholic steatohepatitis [NASH], etc.), and important physiological traits have been identified through GWAS.^4^, ^43^, ^46^ GWAS have been used to elucidate the mechanisms underlying interindividual differences in drug response, which may improve patient prognosis, prevent serious adverse events, and reduce treatment costs.^47^ Furthermore, the fact that GWAS data are derived from human models, rather than animal models, makes it more reliable than nongenomics preclinical target identification experiments for drug target identification and validation.^48^, ^49^

However, 80−90% of phenotype‐associated variants identified by GWAS are present in noncoding regions (e.g., introns, ncRNAs, antisense, enhancers, or insulator regions),^50^ which poses a challenge to the functional interpretation of their proximal regulatory mechanisms, target genes, and the activity of the relevant cell types. And similarly limits global genome analysis for drug discovery, and so attention is turning to sequencing (exome and genome) studies.^51^, ^52^

### 2.2 Whole‐exome sequencing (WES)

WES targets the coding regions (exomes) of all known human nuclear genes and is a highly efficient method for detecting disease‐causing variants,^38^, ^53^, ^54^, ^55^ primarily for disease gene identification and clinical diagnosis. WES can add value to the healthcare system by delivering patient‐oriented care, predicting future medical needs and avoiding unnecessary interventions.^54^ Compared with GWAS, WES is more advantageous in identifying coding and splice‐site mutations,^56^ such as identifying LOF/gain‐of‐function (GOF) mutations and human knockouts (KOs) in closely related populations.^36^ In fact, WES is unable to sequence the entire exome due to a variety of factors such as the lack of capture probes and their sensitivity to guanine‐cytosine content, varying exon sizes, and other structural variants such as inversions or variants in regulations or intronic regions.^38^, ^55^, ^57^

### 2.3 Whole‐genome sequencing (WGS)

The advent of WGS has overcome the limitations of WES to some extent.^58^, ^59^ WGS not only sequences exomes more accurately but also has advantages in detecting large (>50 bp) insertion deletions, copy number variations, and chromosomal rearrangements. Additionally, WGS is able to detect noncoding regions with appropriate coverage. As a result, WGS is emerging as a more comprehensive method for short‐read‐long next‐generation sequencing.^38^, ^60^, ^61^ WGS allows for simultaneous genetic diagnostics and pharmacogenetic analyses, including rare/novel variants, to be performed in a single assay.^38^, ^61^ Using this new personalized therapy to fine‐tune cellular functions or to target specific targets in noncoding regions could yield tremendous results, providing us with new powerful tools to intervene and treat human diseases.^62^

The completion of the Human Genome Project and rapid advances in histological technologies have enabled precise detection of changes in the genome, transcriptome, and proteome.^39^ Genomics‐based next‐generation approaches have several advantages over conventional approaches. While traditional drug discovery methods usually rely on researchers’ assumptions and a priori knowledge, WES, WGS, and GWAS are not limited by a priori knowledge. They allow for comprehensive genomic analysis, which can lead to the discovery of previously undiscovered disease‐related genetic variants and searching for disease‐related therapeutic targets, which greatly enriches the drug targets.^36^, ^37^, ^38^, ^39^ These are also useful for evaluating the efficacy and safety of primary indication targets, as well as identifying additional disease indications to help plan drug development priorities.^49^ In conclusion, the combination of WES, WGS, and GWAS provides an effective strategy for the discovery of new therapeutic targets and the association of old targets with new diseases, and is beginning to be incorporated into the field of pharmacogenetics. Pharmacogenetics represents a major driver of precision medicine and holds the promise of tailoring medical therapies to the pharmacogenetic predispositions of an individual, leading to improved outcomes and reduced costs.^38^, ^39^

## 3 DRUG DEVELOPMENT OF TARGETS BASED ON GWAS, WGS, AND WES DISCOVERY AND VALIDATION

**FIGURE 1:**
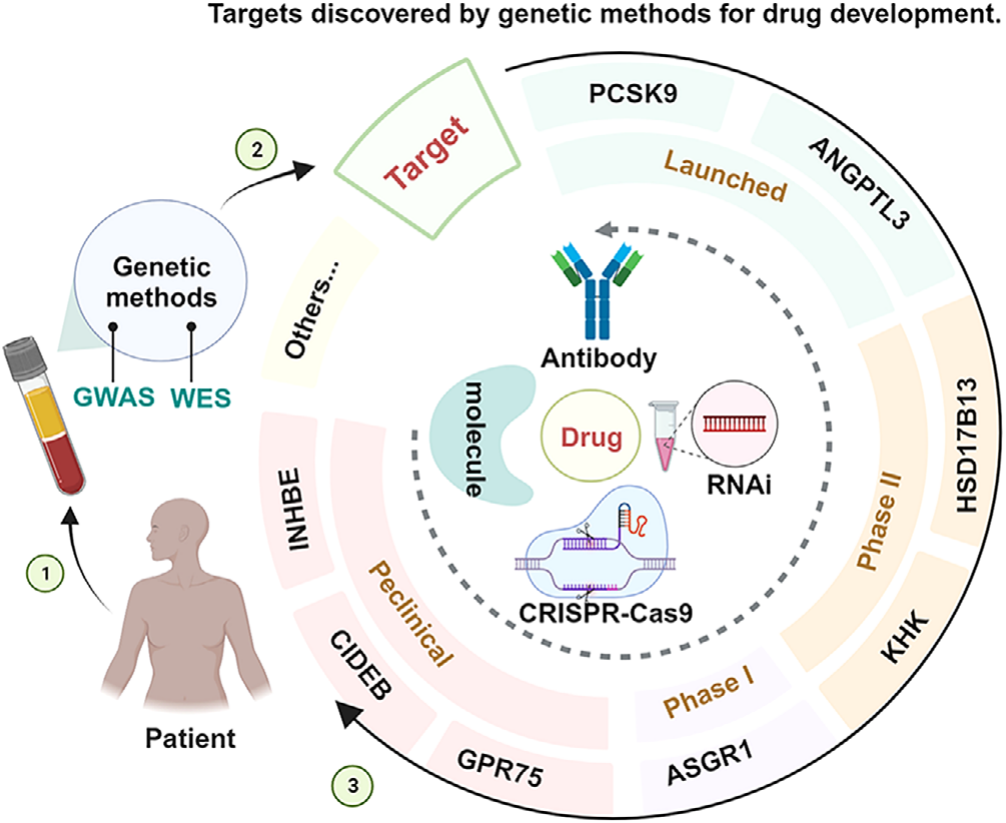
Progress in drug discovery based on targets with favorable LOF variants identified by WES, WGS, and GWAS. Targets primarily include PCSK9, ANGPTL3, HSD17B13, KHK, ASGR1, GPR75, CIDEB, and INHBE. Drug types primarily include small molecules, monoclonal antibodies, RNAi, and CRISPR gene editing therapies. The timeline is determined by the status of the target's current investigational drugs.

WES, WGS, and GWAS have identified several disease‐related gene targets, successfully promoting the study of the biological functions of the targets and the development of their targeted drugs. Moreover, GWAS can associate potential targets with multiple diseases and is expected to suggest new indications for drugs that have been approved or are in clinical trials, with the effect of drug repurposing (for the treatment of other diseases genetically related to the same target).^32^, ^37^, ^63^, ^64^ For example, *FGFR* tyrosine kinase inhibitors (initially developed as anticancer drugs) were later used for chondrodysplasia when it was discovered that GOF mutations in the *FGFR3* gene were also responsible for bone disease.^7^ Similarly, IL‐17A signaling inhibitors originally developed for psoriasis, rheumatoid arthritis, and uveitis have been tested and approved for use in ankylosing spondylitis based on GWAS findings.^7^ Currently, some large pharmaceutical companies have already paid attention to the development of disease genetics research and started to translate their findings into new drug development targets, such as Regeneron Pharmaceuticals Inc and Amgen Inc. In this review, we will focus on some of the targets that have a reduced risk of disease development due to LOF mutations in some genes (Figure 1). We hope to provide the theoretical basis for the research of related targets to promote the research and development of new drugs and drug repurposing. In particular, we hope to develop drugs by mimicking the natural disease‐protective effects of rare LOF variants.

### 3.1 Proprotein convertase subtilisin kexin/type 9

#### 3.1.1 Structure and function of PCSK9

PCSK9 is a serine protease consisting of four parts: a signal peptide, a prestructural domain, a catalytic structural domain, and a C‐terminal cysteine/histidine‐rich structural domain,^65^, ^66^ which is mainly produced by the liver and secreted into the bloodstream.^67^ In addition, there is low expression in the intestinal tract, central nervous system, and renal mesenchymal stromal cells.^67^, ^68^ The link between PCSK9 and cholesterol metabolism was first identified in PCSK9‐overexpressing wild‐type mice,^67^ which had increased plasma triglyceride (TG) and cholesterol‐rich LDL (LDL‐C) levels, whereas hepatic low‐density lipoprotein receptor (LDLR) was virtually absent.^67^ In contrast, *PCSK9* KO mice had reduced plasma cholesterol levels and increased hepatic LDLR.^67^ The understanding of the physiological function of PCSK9 in humans initially arose from the discovery that functional mutations in the *PCSK9* gene resulted in dominant familial hypercholesterolemia (FH).^21^ Abifadel et al.^21^ identified a natural mutant of PCSK9 in patients without mutations in the *LDLR* or *apolipoprotein B* genes, which showed severely high LDL‐C levels. Zhao et al.^69^ reported the presence of two inactivating mutant heterozygotes for PCSK9 in a healthy woman, leaving her with no immunodetectable circulating PCSK9 concentrations and low plasma LDL‐C levels. Separately, DNA sequencing of the *PCSK9* gene in her children similarly revealed LOF, resulting in inhibition of autocatalytic cleavage and secretion of PCSK9.^69^ The clinical benefit of LOF variants in PCSK9 was first described following the sequencing of PCSK9 in African American individuals with hypocholesterolemia from the Dallas Heart Study.^70^, ^71^ Piper et al.^72^ resolved the crystal structure of PCSK9 for the first time and demonstrated that PCSK9 can act by binding to LDLR, providing important support for the development of PCSK9 inhibitors. Several PCSK9 mutations (E32K, L108R, S127R, D374Y, Y142X, C679X, R46L, etc.) were found to be causally associated with lipid metabolism.^21^, ^73^, ^74^, ^75^, ^76^, ^77^, ^78^ WES of university students from Uyghur and other ethnic groups in Xinjiang, China, revealed that PCSK9 LOFs (E144K and C378W) were associated with low plasma levels of LDL‐C.^79^ With the increasing sophistication of genetic testing methods (e.g., WES and WGS), multiple GOF or LOF mutations in PCSK9 have been found to be associated with hypercholesterolemia or hypocholesterolemia.^67^, ^76^, ^80^, ^81^

**FIGURE 2:**
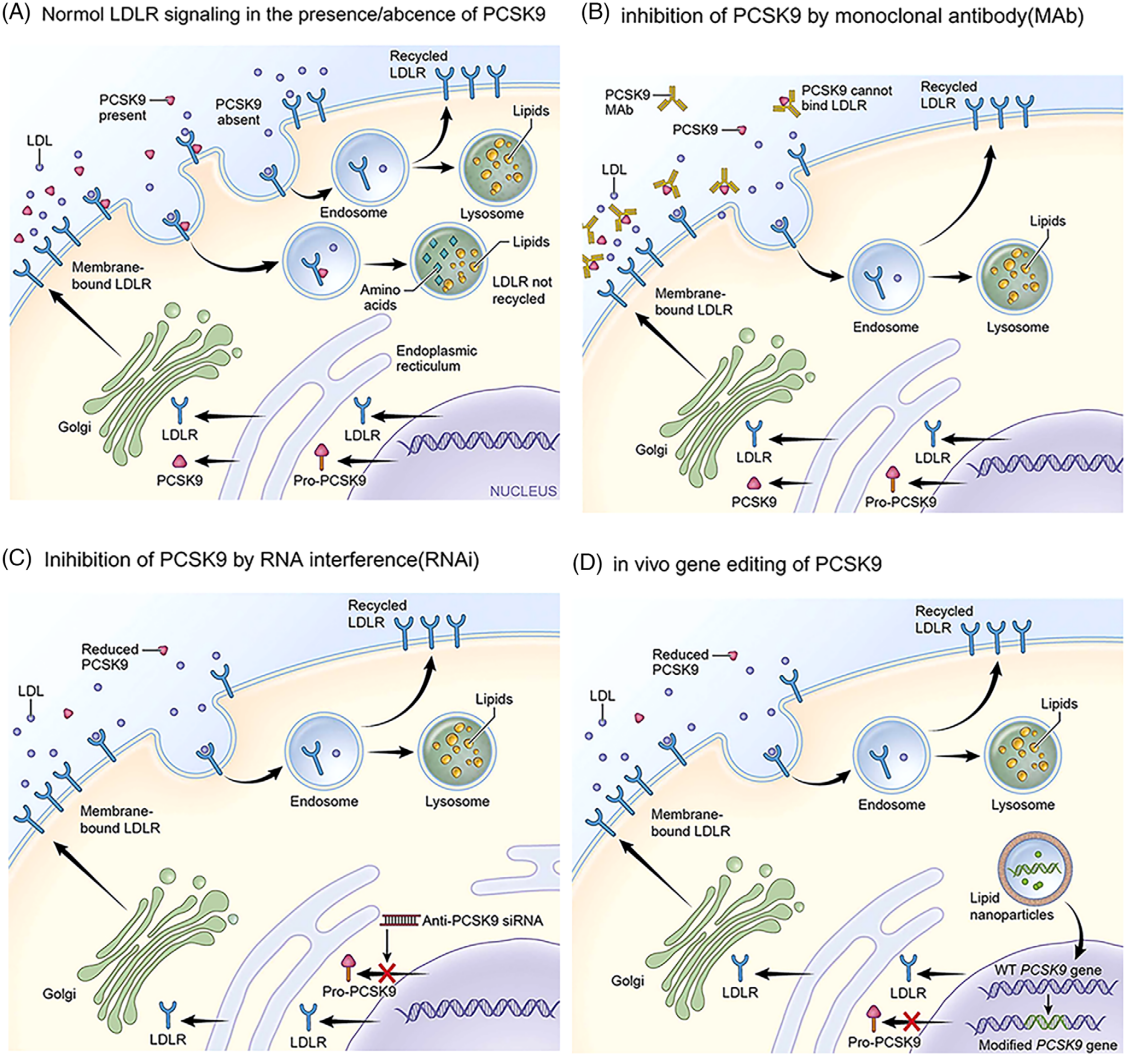
Biological mechanism of PCSK9 and targeted inhibition strategy. (A) When PCSK9 is present, circulating PCSK9 binds to LDLR and LDL particles through its catalytic structural domains, sequestering the LDLR for degradation in the lysosome, which in turn upregulates LDL‐C levels. When PCSK9 is not present, the LDLR loops multiple times.^68^ (B) Monoclonal antibodies (e.g., alirocumab) that bind to and inhibit soluble PCSK9 in plasma, preventing PCSK9 from binding to LDLR and causing LDLR to be degraded by the lysosome.^68^ (C) RNAi reagents (e.g., inclisiran) that prevent successful translation of PCSK9 mRNA, thereby reducing the amount of mature PCSK9 bound to LDLR.^68^ (D) In vivo gene editing of PCSK9 to induce host cell DNA double‐strand breaks to reduce PCSK9 gene expression.^68^ Copyright (2023), with permission from Elsevier. PCSK9, proprotein convertase subtilisin kexin/type 9; LDLR, low‐density lipoprotein receptor; LDLR, low‐density lipoprotein receptor.

It is currently believed that the effects of PCSK9 on lipid metabolism are primarily mediated through the regulation of hepatic LDLR levels (Figure 2A).^68^, ^82^

Studies have shown that PCSK9‐mediated increases in LDL‐C are strongly associated with the progression of cardiovascular diseases such as coronary heart disease. In addition, PCSK9 affects blood lipid levels by promoting hepatic lipogenesis, which indirectly contributes to the development of atherosclerosis (AS).^83^, ^84^, ^85^ PCSK9 also induces the expression of pro‐inflammatory cytokines that mediate the inflammatory response,^66^, ^84^, ^86^ which may be related to the C‐terminal cysteine/histidine‐rich structural domain of the PCSK9 protein.^86^ PCSK9 also binds to CD36 to promote platelet activation and thrombosis and to reduce fatty acid uptake and TG accumulation in tissues.^86^ A significant correlation has been found between PCSK9 and glucose metabolism,^87^ but studies linking *PCSK9* LOF variants to the risk of developing diabetes are conflicting.^88^, ^89^ PCSK9 may also be involved in the pathogenesis of fatty liver disease. For example, *PCSK9* rs11591147 (p.R46L) LOF has a protective effect on hepatic steatosis, NAFLD/NASH, and fibrosis.^90^, ^91^ Besides, PCSK9 has been implicated in various diseases such as cancer biology and neurological disorders.^92^, ^93^ It has been shown that *PCSK9* LOF prevents the development of abdominal aortic aneurysm in preclinical mouse models.^94^ Furthermore, several experiments have demonstrated that PCSK9 is associated with cancers.^95^, ^96^ In conclusion, PCSK9 can play pleiotropic roles in various biological pathways, but the most widely studied remains its function in maintaining cholesterol homeostasis.

#### 3.1.2 Drug development of PCSK9

Genetic studies have shown that carrying the *PCSK9* LOF allele is associated with low LDL‐C levels and that carriers live long and healthy lives, suggesting that the mutation is harmless, which will greatly facilitate research on PCSK9 inhibitors. Current PCSK9 inhibition strategies mainly include blocking PCSK9 synthesis (e.g., antisense oligonucleotide [ASO] or small interfering RNA [siRNA] that silence *PCSK9* mRNA and CRISPR–Cas9 gene editing therapy) and targeting PCSK9 protein (small molecule, mimetic peptide, antibody, and vaccine that prevent PCSK9 from binding to the LDLR, etc.) (Figures 2B, C, and D).^68^

Two monoclonal antibodies (alirocumab,^97^ evolocumab^98^) and one siRNA (inclisiran^99^) targeting PCSK9 have been approved and marketed globally (Table 1). They have potent lipid‐lowering effects in preclinical and clinical trials and are safe and well‐tolerated. This further validates the feasibility of PCSK9 as a novel lipid‐lowering target. With the introduction of three new drugs, the global development of PCSK9 inhibitors is at an all‐time high. Although PCSK9 inhibitors also have application value in the treatment of some tumors, viral infections, and other diseases, they are still mainly used in the treatment of hyperlipidemia, AS, and related ischemic cardiovascular diseases.^100^ Therefore, PCSK9 inhibitors have multiple indications that need to be investigated in the future, and the application prospect is promising. According to incomplete statistics, there are currently about 90 PCSK9 inhibitors in the research phase worldwide. In this review, we will focus on summarizing the drugs and technologies that have entered clinical phases II and III and are still in the pipeline (Table 1).

**TABLE 1:** Marketed and phase II/III investigational drugs/technologies that target PCSK9.

Drug name	Originator company	Status	Target disease	References
Evolocumab	Amgen Inc	Launched	Mixed dyslipidemia; primary HC; HoFH; HeFH; ASCVD	^25^, ^101^, ^102^, ^103^, ^104^, ^105^, ^106^, ^107^
Alirocumab	Regeneron Pharmaceuticals Inc	Launched	Mixed dyslipidemia; primary HC; ASCVD; FH; HC; Lipid metabolism disorder	^24^, ^25^, ^102^, ^103^
Inclisiran	Alnylam Pharmaceuticals Inc	Launched	Primary HC; FH; HC; ASCVD; HeFH	^108^, ^109^, ^110^
Tafolecimab/IBI306	Adimab LLC	Registered	HLP; HC	^103^, ^111^, ^112^
Ongericimab/JS002	Shanghai Junshi Biosciences Co Ltd	Preregistration	BCA; HCC; HLP; HC; lipid metabolism disorder; lung tumor; metastasis	^103^, ^113^
Ebronucimab/AK102	Akeso Biopharma Inc	Preregistration	FH; HLP; HC	^103^, ^114^, ^115^
Recaticimab/SHR‐1209	Suzhou Shengdiya Biomedical Co Ltd	Preregistration	FH; HC; HLP	^67^, ^116^, ^117^, ^118^
Lerodalcibep/LIB‐003	Adnexus Therapeutics Inc	Phase 3 Clinical	CVD; FH; HC; stroke	^119^, ^120^
Enlicitide chloride/MK‐0616	Merck & Co Inc	Phase 3 Clinical	CAD; HC	^121^, ^122^, ^123^, ^124^
AZD‐8233	Ionis Pharmaceuticals Inc	Phase 2 Clinical	HC; HLP; Lipid metabolism disorder; NIDDM	^125^
CVI‐LM001	CVI Pharmaceuticals Ltd	Phase 2 Clinical	HLP; HC	^126^
Cepadacursen sodium/Civi‐007	Civi Biopharma, Inc	Phase 2 Clinical	HC	^127^

Abbreviations: AS, atherosclerosis; ASCVD, atherosclerotic cardiovascular disease; BCA, bladder cancer; CAD, coronary atherosclerotic heart disease; CVD, cardiovascular disease; FH, familial hypercholesterolemia; HC, hypercholesterolemia; HCC, hepatocellular carcinoma; HeFH, heterozygous familial hypercholesterolemia; HLP, hyperlipidemia; HoFH, homozygous familial hypercholesterolemia.

In summary, the discovery of the *PCSK9* gene and related LOF mutations lays the foundation for new therapeutic strategies targeting PCSK9 for the treatment of hyperlipidemia and ASCVD. The efficacy and safety of marketed PCSK9 inhibitors confirm that PCSK9 is an excellent therapeutic target for cardiovascular and related diseases. It also validates that targets based on human genetics are more conducive to clinical translation, indicating the importance of WES, WGS and GWAS in drug discovery and development, as they point to pharmacologic inhibition of PCSK9 as a safe strategy to prevent or treat CAD. In addition, PCSK9 inhibition is rapidly evolving as a potential therapeutic mechanism for various ailments, including some unrelated to cholesterol and lipid metabolism. PCSK9 inhibitors remain one of the hotspots for future research, especially for nucleic acid‐based therapies,^128^, ^129^ for example, siRNA, ASO, and CRISPR base editing. siRNA technology may extend the duration of action and may circumvent drug adherence problems.^130^ Of course, it remains to be determined whether gene editing tools will be used clinically to reduce the risk of LDL‐C and cardiovascular disease due to safety and ethical concerns.^131^ In addition, vaccination makes one of the novel therapies to inhibit PCSK9.^130^

### 3.2 Angiopoietin‐like protein 3

#### 3.2.1 Structure and function of ANGPTL3

Angiopoietin‐like protein 3 (ANGPTL3) is a secreted protein consisting of a signal peptide sequence, an N‐terminal helical structural domain (CCD), and a C‐terminal globular fibrinogen homologous structural domain.^132^, ^133^, ^134^ ANGPTL3 is expressed predominantly in the liver and secreted into the circulatory system,^132^, ^133^, ^134^ where its expression is activated by oxysterol‐stimulated hepatic X receptors.^135^

Koishi et al.^136^ found the plasma TG and free fatty acid (FFA) were significantly lower in KK/San mice than those of KK‐obese mice. This study establishes the link between ANGPTL3 and lipoprotein metabolism,^136^ indicating that the reduced ANGPTL3 expression reduces the risk of hyperlipidemia and ASCVD. GWAS has identified SNPs in the vicinity of the ANGPTL3 locus that have a strong effect on lipid metabolism, thus confirming the prominent role of ANGPTL3 in lipid metabolism.^137^, ^138^ Romeo et al.^139^ found that mutations in ANGPTL3 were significantly associated with human TG levels. Furthermore, WES of more than 180,000 individuals with *ANGPTL3* LOF variants showed that heterozygous carriers of *ANGPTL3* LOF mutations had approximately 34% lower odds of CAD than noncarriers,^139^ suggesting an association between CAD and plasma levels of ANGPTL3. By sequencing the ANGPTL3 exons of 58,335 participants in the DiscovEHR human genetics study, Dewey et al.^140^ identified 13 different LOF variants of *ANGPTL3*, the presence of which was associated with a 41% lower risk of CAD in 13,102 patients with CAD and 40,430 controls. In addition, carriers of the *ANGPTL3* LOF variant were found to have 27% lower levels of TG, 9% lower levels of LDL‐C, and 4% lower levels of high‐density lipoprotein cholesterol (HDL‐C) compared with noncarriers.^141^ Similar results were reported by Stitziel et al.^142^ Musunuru et al.^143^ found by WES that two mutations (S17X and E129X) in human ANGPTL3 resulted in decreased plasma LDL‐C, TG, and HDL‐C levels, and also minimized the rate of very low‐density lipoprotein (VLDL)‐apo B production and increased the rate of LDL‐apo B catabolism. Robciuc et al.^144^ reported that *ANGPTL3* LOF carriers exhibit low plasma FFA levels and strong insulin sensitivity. ASO targeting *ANGPTL3* mRNA reduces lipid levels and decreases ASCVD progression in mice.^145^ Importantly, patients with ANGPTL3 deficiency did not have hepatic steatosis.^146^ The data from the above studies emphasize the importance of ANGPTL3 genetic variation in regulating lipid metabolism in humans and support the idea that genetic inactivation or low levels of ANGPTL3 are essential in lowering lipids and reducing the risk of ASCVD.

The mature CCD of ANGPTL3 binds lipoprotein lipase (LPL) and endothelial lipase (EL) and efficiently inhibits their catalytic activities.^133^, ^134^, ^147^, ^148^, ^149^ The bioactivity of LPL is a critical factor in the rate of removal of circulating TG‐rich lipoproteins (TRL) and plays a major role in TG and VLDL metabolism.^133^, ^150^ EL is a key enzyme in the regulation of HDL‐C metabolism.^151^ ANGPTL3 induces lipolysis in adipose tissue, leading to the release of FFA and glycerol from adipocytes, which results in increased hepatic VLDL synthesis.^134^, ^149^

**FIGURE 3:**
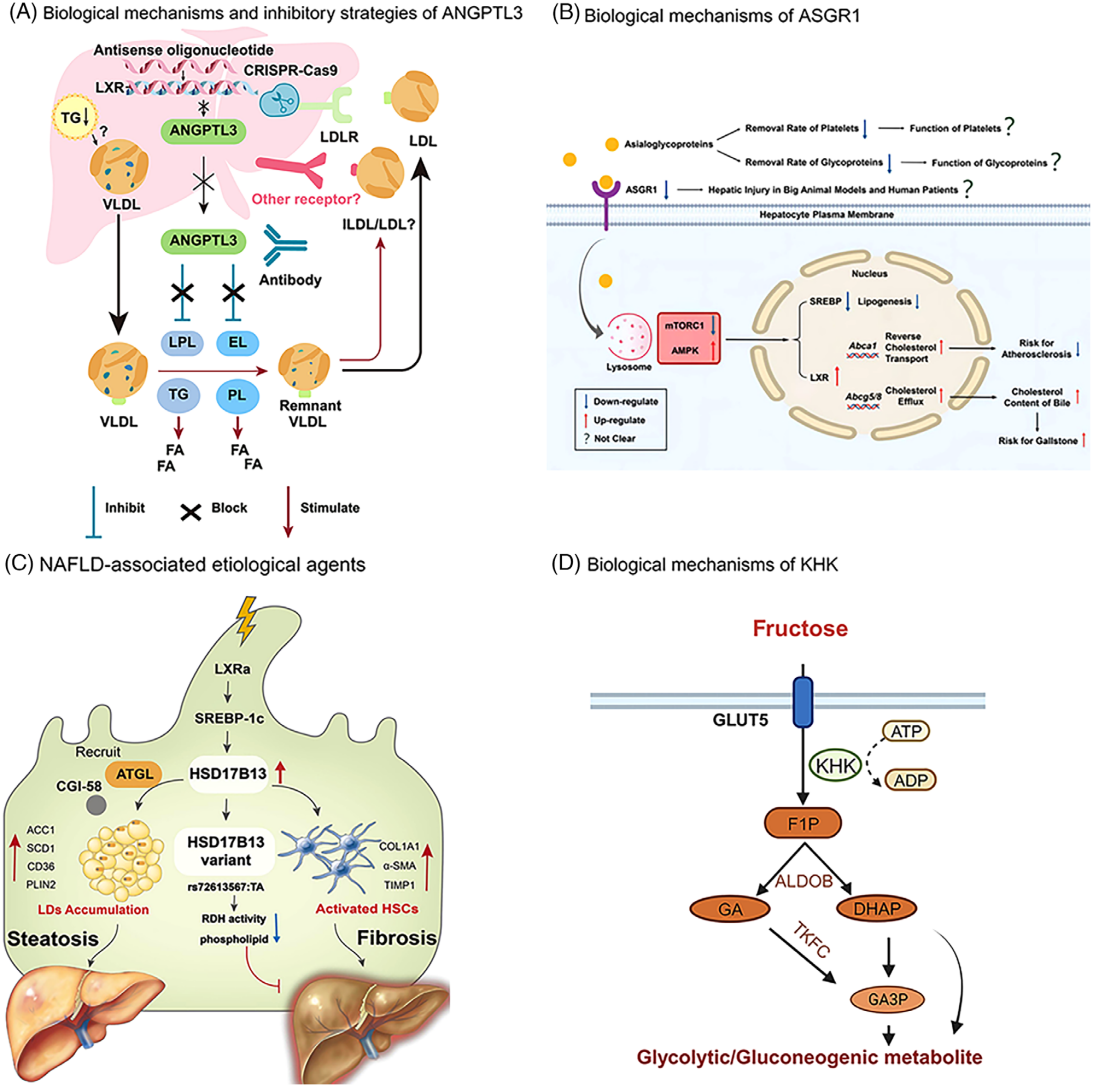
Biological mechanism of ANGPTL3, ASGR1, HSD17B13, and KHK and targeted inhibition strategy. (A) Role of ANGPTL3 in lipoprotein metabolism and strategies for its inhibition.^153^ Upon inhibition of ANGPTL3, TG‐rich lipoproteins was hydrolyzed by free lipoprotein lipase, thereby reducing TG and other cholesterol levels.^153^ Copyright (2023), with permission from Elsevier. (B) ASGR1 regulates lipid metabolism.^185^ Downregulation of ASGR1 leads to AMPK activation and mTORC1 inhibition, which results in downregulation of SREBP and upregulation of LXR and triggers a series of responses in cholesterol metabolism, ultimately leading to a reduction in circulating cholesterol levels.^185^ (C) Role of HSD17B13 in NAFLD progression.^208^ HSD17B13 can be upregulated by LXRα/SREBP‐1c, which then recruits adipose triglyceride lipase (ATGL) and comparative gene identification‐58 (CGI‐58) to induce LD aggregation and regulate the dynamics of LDs. The HSD17B13 variant loses RDH activity and has lower phospholipid levels, showing preventive effects against NAFLD.^208^ Copyright (2023), with permission from Elsevier. (D) In vivo metabolism of fructose catalyzed by KHK. Fructose is phosphorylated by KHK to F1P, which is then catabolized by ALDOB to DHAP and glyceraldehyde. Glyceraldehyde is phosphorylated to GA3P by TKFC. DHAP and GA3P enter the glycolysis/glycogen pathway at the level of trisaccharide phosphate.^245^ Created with BioRender.com. ANGPTL3, angioprotein‐like 3; CRISPR, clustered regularly interspaced short palindromic repeats; LDL, low‐density lipoprotein; HDL, high‐density lipoprotein. ASGR1, asialoglycoprotein receptor 1; AMPK, AMP‐activated protein kinase; mTORC1, target of rapamycin complex 1; SREBP, sterol regulatory element binding protein; LXR, liver X receptor; HSD17B13, 17‐β‐hydroxysteroid dehydrogenase 13; NAFLD, nonalcoholic fatty liver disease; KHK, ketohexokinase; F1P, fructose‐1‐phosphate; ALDOB, aldolase B; DHAP, dihydroxyacetone phosphate; GA3P, glyceraldehyde 3‐phosphate; TKFC, trisaccharide kinase.

The detailed mechanisms by which ANGPTL3 regulates LDL‐C levels are poorly understood, and it is initially believed that there are two pathways of LDL‐C regulation by ANGPTL3.^132^, ^152^ One is independent of LDLR (in the absence of LDLR)^152^ and may control LDL‐C levels by regulating EL‐dependent VLDL clearance (Figure 3A).^153^, ^154^ The other is through LDLR independently of the EL pathway.^132^ Data from preclinical and clinical studies of evinacumab, a monoclonal antibody to ANGPTL3, provide additional support for a pathway independent of LDLR.^155^ Therefore, inhibition of ANGPTL3 is expected to produce better therapeutic effects in patients with LDLR deficiency, such as HoFH. In addition, ANGPTL3 has a regulatory effect on TG and HDL‐C (Figure 3A).^148^, ^153^ ANGPTL3 also affects glucose and insulin metabolism.^142^, ^149^, ^156^ *ANGPTL3* LOF mutations reduce risk for developing Type 2 Diabetes (T2D) and coronary heart disease.^149^ Furthermore, ANGPTL3 expression is elevated in the livers of NAFLD patients,^149^, ^157^ and its inhibitors have the potential to mitigate the progression of NAFLD and provide promising therapeutic approaches for NAFLD. In addition, it has been shown that ANGPTL3 is highly expressed in hepatocellular carcinoma,^158^ ovarian cancer,^159^ and oral squamous cell carcinoma,^149^ which may be related to the fact that ANGPTL3 induces cancer‐associated fibroblasts differentiation.^158^, ^160^

#### 3.2.2 Drug development of ANGPTL3

Even with some recent therapeutic advances (statins, PCSK9 inhibitors, etc.), the need for low LDL‐C in patients with very high baseline LDL‐C levels (e.g., patients with FH, and especially HoFH) remains unmet.^161^ In addition, some patient groups have residual cholesterol and TRL‐induced ASCVD risk dependent. Therefore, there remains an urgent need to develop additional lipid‐lowering agents independent of LDLR activity that can appropriately lower cholesterol levels in TRL while lowering LDL‐C levels in order to address the lipid‐lowering needs that remain unmet by the maximal tolerated dose of current lipid‐lowering therapies. ANGPTL3, which can affect lipid levels in a variety of ways, has emerged as an emerging lipid‐lowering therapeutic target for scientists.

Based on the above studies and the favorable consequences of the lack of ANGPTL3, interest in ANGPTL3 as a therapeutic target has been stimulated. Several research techniques (including monoclonal antibodies against ANGPL3 protein, ASO and siRNA drugs targeting ANGPTL3 mRNA, clustered regularly interspaced short palindromic repeats (CRISPR) gene editing therapies, vaccines, and small molecules, etc.) have been proposed to inhibit ANGPTL3 (Figure 3A),^153^, ^162^, ^163^ and have yielded encouraging results.

One ANGPTL3 inhibitor is currently approved for marketing, the human monoclonal antibody evinacumab developed by Regeneron.^141^, ^164^ Currently, evinacumab is used primarily in patients with HoFH (both children and adults over the age of 5 years),^165^ and several studies have validated the long‐term safety and efficacy of evinacumab.^141^ Second, there are several ANGPTL3 inhibitors in the development stages (Table 2), among which the more hotly researched ones are ASO and siRNA drugs targeting ANGPTL3 mRNA, such as ARO‐ANG3 and Vupanorsen.^166^, ^167^

**TABLE 2:** Investigational drugs/technologies targeting ANGPTL3.

Drug name	Originator company	Status	Target disease	References
ARO‐ANG3	Arrowhead Pharmaceuticals Inc	Phase 2 clinical	HC; FH; Lipid metabolism disorder	^168^, ^169^
Vupanorsen	Ionis Pharmaceuticals Inc	Phase 2 clinical	HTG	^148^, ^170^
LNA‐043	Novartis Pharmaceuticals Corp	Phase 2 clinical	OA	^171^, ^172^
LY‐3561774	Dicerna Pharmaceuticals Inc	Phase 2 clinical	CVD; Lipid metabolism disorder	^173^, ^174^
LY‐3475766	Eli Lilly & Co	Phase 1 clinical	AS	^175^
VERVE‐101	Verve Therapeutics Inc	Phase 1 clinical	FH; CAD; AS; MI	^176^
JS‐401	Shanghai Junshi Biosciences Co Ltd	Preclinical	HLP, Metabolic disorder	^177^
VERVE‐201	Verve Therapeutics Inc	Preclinical	FH	^178^
CTX‐310	CRISPR Therapeutics AG	Preclinical	CVD	^179^
SR‐045	Suzhou Ribo Life Science Co Ltd	Preclinical	HLP	^180^

Abbreviations: HTG, hypertriglyceridemia; MI, myocardial infarction; OA, osteoarthritis.

Based on the LDL‐C regulatory mechanism of ANGPTL3 independent of LDLR, it is expected to make up for the inadequacy of current PCSK9 inhibitors in lowering LDL‐C and TG to address the residual risk of ASCVD. Inhibition of ANGPTL3 also reduces TG levels, achieving a multifaceted lipid‐lowering effect. In conclusion, inhibition of ANGPTL3 expression modulates multiple biological effects such as glucose uptake, insulin sensitivity, LDL/VLDL uptake, and TG/TRL metabolism.^157^ Clinical trials have now demonstrated the effective role of ANGPTL3 inhibition in lipid‐lowering. In the future, ANGPTL3 is expected to be a very promising novel therapeutic target for lipid metabolism and is expected to further reduce the risk of ASCVD. In addition, *ANGPTL3* LOF mutations prevent CAD without serious adverse health consequences and are expressed predominantly in hepatocytes, several features that make nucleic acid therapy potentially more effective than monoclonal antibodies. In particular, inhibition of ANGPTL3 by siRNA may be a future panacea against hypertriglyceridemia in a variety of dyslipidemia conditions,^181^ including residual LDL‐C elevation in FH.^135^, ^166^, ^182^ Similarly, ANGPTL3 is an attractive target for in vivo gene editing. Furthermore, anti‐dyslipidemia vaccine therapies targeting ANGPTL3 will be a hot research topic.^183^ Of course, the long‐term safety and cost‐effectiveness of these agents remain to be demonstrated in ongoing and future clinical trials.^137^

### 3.3 Asialoglycoprotein receptor 1

#### 3.3.1 Structure and function of ASGR1

Asialoglycoprotein receptor 1 (ASGR1) is mainly expressed on liver parenchymal cell membranes^184^ and can mediate endocytosis and lysosomal degradation of glycoproteins with exposed terminal galactose or N‐acetylgalactosamine (GalNAc) residues.^185^ It was shown that the integrity of the C‐terminal Ca^2+^‐dependent carbohydrate‐recognizing structural domain of ASGR1 is critical for its ability to reduce cell surface LDLR levels.^186^ Early studies on ASGR1 focused on its efficient delivery of GalNAc‐ASO and GalNAc‐siRNA‐coupled drugs as liver‐specific receptors.^187^, ^188^

In 2016, Nioi et al.^189^ identified a deletion mutation in intron 4 of *ASGR1* with a segment of 12 base pairs in length by sequencing the Icelandic genome, which resulted in a reduced incidence of cardiovascular disease. The same study also found that another LOF variant of *ASGR1* (W158X) was associated with reduced non‐HDL cholesterol levels.^185^, ^189^ Subsequently, humans carrying the *ASGR1* LOF variant allele were found to have lower serum levels of non‐HDL cholesterol and a lower risk of CAD and myocardial infarction, compared with noncarriers.^189^, ^190^ Some studies have shown that lowering ASGR1 increases LDLR, which in turn lowers blood cholesterol levels.^185^ For example, Xie et al.’s study of ASGR1‐deficient pigs found that ASGR1 deficiency leads to downregulation of HMGCR to reduce de novo cholesterol biosynthesis in the liver.^185^, ^191^ In addition, hepatic LDLR is upregulated to increase LDL‐C clearance.^185^, ^191^

In 2022, Wang et al.^184^ discovered the detailed mechanisms by which the ASGR1 pathway regulates lipid metabolism through multiple studies (Figure 3B). Desialylate glycoproteins bind to ASGR1 and are delivered to the lysosome, causing these glycoproteins to digest and release amino acids, which activate lysosomal mechanistic target of rapamycin complex 1 (mTORC1) and block AMP‐activated protein kinase (AMPK) to increase Breast Cancer 1 protein (BRCA1)/BRCA1‐associated RING domain protein 1 (BARD1) and mediate liver X receptor (LXR) degradation.^184^, ^185^ In addition, several studies have shown that the effect of ASGR1 on blood cholesterol is related to the role of sterol regulatory element binding protein (SREBP) in the cell nucleus.^185^ Xu et al.^192^ found reduced SREBP in *ASGR1* KO HepG2 cells and mouse liver tissue. Similar findings were obtained in a study by Wang et al.^184^ Meanwhile, that study, by targeting and inhibiting the expression of ASGR1 using siRNA interference, observed that mice had lower blood cholesterol levels, increased bile cholesterol levels, and even ameliorated AS induced by a high‐fat diet in the mice.^184^

ASGR1 plays a key role in a variety of pathophysiological processes, such as removing salivary acidified platelets, inhibiting metastasis of hepatocellular carcinoma, and clearing LDL and celiac remnants.^193^, ^194^ In addition, hepatic ASGR1 was shown to bind the N‐terminal structural domain of the SARS‐CoV‐2 spiking glycoprotein and the ACE2 receptor‐binding domain,^186^ protecting the virus from certain neutralizing antibodies. Shi et al.^195^ suggested that ASGR1 regulates local and systemic inflammation and lethality of liver injury by enhancing monocyte‐to‐macrophage differentiation. The hepatic specificity of ASGR1 reduces the theoretical possibility that ASGR1 inhibition causes multiorgan adverse effects, making ASGR1 more favorable for therapeutic development.

#### 3.3.2 Drug development of ASGR1

Early studies on ASGR1 focused on its efficient delivery of GalNAc‐ASO and GalNAc‐siRNA‐coupled drugs as liver‐specific receptors.^187^ Currently, five siRNA drugs based on the mechanism of ASGR1 specific recognition of GalNAc for targeted delivery are approved and marketed worldwide (Table 3).

**TABLE 3:** Marketed GalNAc‐siRNA coupling drugs.

Drug name	Originator company	Status	Target disease	References
Givosiran	Alnylam Pharmaceuticals Inc	Launched	AHP	^196^
Lumasiran	Alnylam Pharmaceuticals Inc	Launched	PH1	^197^
Inclisiran	Alnylam Pharmaceuticals Inc	Launched	Primary HC; FH; HC; ASCVD; HeFH	^108^, ^109^, ^110^
Vutrisiran	Alnylam Pharmaceuticals Inc	Launched	Familial amyloid neuropathy; Stargardt disease	^198^
Rivfloza	Dicerna Pharmaceuticals Inc	Launched	PH1; End stage renal disease	^199^

Abbreviations: AHP, acute hepatic porphyria; PH1, primary hyperoxaluria type 1.

In recent years, researchers have found that ASGR1 inhibitors reduce Hypercholesterolemia by inhibiting cholesterol biosynthesis and promoting cholesterol externalization and internal excretion.^185^ In addition, ASGR1 inhibitors can exhibit synergistic effects with atorvastatin or ezetimibe in lipid lowering.^200^ This may provide a theoretical basis for the future use of ASGR1 inhibitors in combination with other cholesterol‐lowering drugs. In conclusion, therapies targeting ASGR1 represent a novel treatment for lowering non‐HDL cholesterol levels and treating cardiovascular disease, particularly CAD.

Drugs and technologies targeting ASGR1 are currently in the development stage, such as Amgen's ASGR1 inhibitor (AS) and AMG 529.^201^ In addition, a study identified natural ASRG1 inhibitors derived from food as potential anti‐hypercholesterolemia drugs by computer simulation.^200^ Compared with PCSK9, less research has been done on ASGR1 as a drug target, suggesting a broader market for the development of new drugs from this target.

Of course, there are potential risks associated with inhibiting ASGR1. For example, by increasing the cholesterol content of bile, the probability of developing gallstones increases.^185^ It may also lead to mild liver injury, as Xie et al. found mild to moderate liver injury in ASGR1‐deficient pigs.^191^ Therefore, future studies of ASGR1 are necessary to elucidate whether its inhibition causes liver injury. In addition, reduced ASGR1 inhibits the removal of old platelets and the production of new platelets, affecting the physiologic function of platelets.^185^

Overall, there are no lipid‐lowering drugs on the market that work by targeting cholesterol degradation or excretion. ASGR1 has great potential as a target for regulating cholesterol efflux and fatty acid synthesis for lipid‐lowering drug development.

### 3.4 17‐β‐Hydroxysteroid dehydrogenase 13

#### 3.4.1 Structure and function of HSD17B13

17‐β‐Hydroxysteroid dehydrogenase 13 (HSD17B13) is a hydroxysteroid dehydrogenase that catalyzes the conversion between 17‐keto and 17‐hydroxysteroids.^202^ It consists of six structural domains: hydrophobic domain (HD), PAT‐like structural domain, cofactor‐binding structural domain (CFB), folding/dimerization structural domain (F/D), catalytic structural domain (CAT), and stability/unknown structural domain (S/U).^203^, ^204^, ^205^ HSD17B13 is highly expressed mainly in mouse and human liver. In addition, human HSD17B13 has been identified as a retinol dehydrogenase (RDH),^206^, ^207^ which catalyzes the conversion of retinol to retinaldehyde in combination with appropriate LD targeting and cofactors and is the rate‐limiting enzyme controlling the biosynthesis of trans‐retinoic acid during NAFLD progression.^208^ It has been shown that HSD17B13 stability, targeting LD, and enzymatic function are mainly influenced by HD and PAT.^203^, ^209^ Of course, CFB and F/D also affect the enzymatic activity of HSD17B13.^203^, ^209^

HSD17B13 expression is upregulated in NAFLD/NASH patients.^210^ In 2014, Su et al.^211^ first reported HSD17B13 as the causative protein of NAFLD. In 2018, Abul‐Husn et al.^212^ performed WES on 46,544 Americans and found that the *HSD17B13* LOF mutation (rs72613567:TA) was associated with chronic liver disease,^213^ risk of progression from steatosis to steatohepatitis, and reduced plasma alanine aminotransferase (ALT) and glutamate aminotransferase (AST) levels, and also revealed that the variant was negatively associated with chronic liver disease in an allelic dose‐dependent manner.^213^ Subsequently, the hepatoprotective effect of the *HSD17B13* rs72613567 variant was confirmed in several independent population‐based genetic studies of chronic liver disease.^214^, ^215^, ^216^, ^217^, ^218^, ^219^ For example, Yang et al.^214^ reported that the *HSD17B13* LOF variant (rs72613567) prevents hepatocellular carcinoma (HCC) development in patients with alcoholic liver disease; Chen et al.^215^ and Kallwitz et al.^216^ reported that genetic variation in the *HSD17B13* gene was associated with a reduced risk of NAFLD in Chinese Han Chinese populations and Hispanics/Latinos; Vilar‐Gomez et al.^217^ reported that the role of *HSD17B13* rs72613567 in reducing the development of NASH and hepatic fibrosis may be influenced by the coexistence of other genetic variants as well as clinical risk factors including diabetes, obesity, and alcohol consumption. In addition, it has been reported that the *HSD17B13* gene variant may be involved in the regulation of T2D‐associated dyslipidemia.^220^

Of course, the relationship between HSD17B13 and NASH risk varies by study. For example, Adam et al.^221^ reported data on mice that, in contrast to results in humans, showed that *HSD17B13* KO induced steatosis and inflammation in male mice. Ma et al.^207^ have since similarly failed to reproduce the protective effect of the *HSD17B13* LOF mutant in human NAFLD in mice, suggesting that the function of HSD17B13 may be affected by differences between species.

The relationship between HSD17B13 and NASH risk varies by study. For example, Adam et al.^221^ reported data on mice that, in contrast to results in humans, showed that HSD17B13 KO induced steatosis and inflammation in male mice. Ma et al.^207^ have since similarly failed to reproduce the protective effect of the *HSD17B13* LOF mutant in human NAFLD in mice, suggesting that the function of HSD17B13 may be affected by differences between species.

The current mechanism of action of HSD17B13 in NAFLD is mainly shown in Figure 3C. Multiple lines of evidence suggest that vitamin A metabolites (retinaldehyde and retinoic acid) play important roles in hepatic mitochondrial fatty acid β‐oxidation, immunomodulation, inhibition of hepatic fibrosis, and insulin resistance,^222^ further correlating the relationship between HSD17B13 and NAFLD.

Studies of *HSD17B13* LOF and siRNA silencing of *HSD17B13* mRNA found that hepatic steatosis was unaffected in subjects, and it was tentatively concluded that the lack of preventive effect of HSD17B13 on NASH and other related diseases is unlikely to be due to reduced metabolism of hepatic fat stores. Thus, further studies are needed to elucidate the role of HSD17B13 in simple steatosis. Luukkonen et al.^223^ indicated that the protection against hepatic fibrosis induced by the human HSD17B13 variant and the mouse *HSD17B13* KO was associated with reduced pyrimidine catabolism at the level of dihydropyrimidine dehydrogenase.

Overall, the regulation of HSD17B13 in hepatic lipid metabolism and the pathogenesis of NAFLD/NASH is still unclear and requires further studies. Furthermore, understanding interspecies differences may be key to unraveling the mechanism of action of human HSD17B13 and advancing its role as a therapeutic target for fatty liver.

#### 3.4.2 Drug development of HSD17B13

Although the results obtained in animal models are inconsistent with those in humans. However, several large clinical and population‐based GWAS have shown a robust and reproducible association between *HSD17B13* gene variants and the natural history of NAFLD/NASH.^224^ Both in vivo and in vitro studies have shown that inhibition of HSD17B13 expression is beneficial for the treatment of NAFLD/NASH and is one of the emerging potential targets for NAFLD/NASH. Although the mechanism of action of HSD17B13 is not fully understood, based on the genetic and preclinical studies mentioned above, researchers have carried out studies on HSD17B13 as a drug target (Table 4), with more hot research on siRNA drugs targeting inhibition of *HSD17B13* mRNA expression.

**TABLE 4:** Investigational drugs/technologies targeting HSD17B13.

Drug name	Originator company	Status	Target disease	References
ARO‐HSD	Arrowhead Pharmaceuticals Inc	Phase 2 clinical	Liver disease; NASH; ALD	^227^, ^228^
FOR‐6219	Hormos Medical Corp	Phase 2 clinical	Endometriosis; Endometrioid carcinoma; Uterine fibroids; Breast tumor	^229^
ALN‐HSD	Regeneron Pharmaceuticals Inc	Phase 2 clinical	NASH	^230^
INI‐822	Inipharm	Phase 1 clinical	Fibrosis; Liver disease; NASH	^231^, ^232^
ION‐455/AZD7503	Ionis Pharmaceuticals Inc	Phase 1 clinical	Steatohepatitis; NAFLD; NASH	^232^, ^233^
RBD1073	Suzhou Ribo Life Science Co Ltd	Preclinical	NASH	^234^
BI‐3231	Boehringer Ingelheim International GmbH	Preclinical	Liver disease; NASH; NAFLD; Liver cirrhosis	^235^

Abbreviations: ALD, alcoholic liver disease; NASH, nonalcoholic steatohepatitis.

Currently, the main siRNA‐based drugs are ARO‐HSD and ALN‐HSD. Among them, ARO‐HSD is a GalNAc‐coupled RNAi therapy developed by Arrowhead that selectively targets *HSD17B13* mRNA, aiming to downregulate the expression of HSD17B13 to obtain a beneficial LOF effect, and is being developed for the treatment of NASH.^225^ ARO‐HSD phase 1/2a results showed that the inhibitory effect of ARO‐HSD on *HSD17B13* mRNA and HSD17B13 protein levels exhibited a dose‐dependent effect in all patients, even more than 90% inhibition of *HSD17B13* mRNA at a dose of 200 mg, and was well tolerated and safe.^225^, ^226^ In addition, ARO‐HSD significantly reduced serum AST and ALT levels.^226^ Consistent with what was observed in *HSD17B13* LOF carriers,^225^ ARO‐HSD did not directly affect hepatic steatosis.

In conclusion, variants of *HSD17B13* LOF are associated with NASH/NAFLD. HSD17B13 based on human genetic studies holds great promise for drug development as a potential therapeutic target for NASH and related liver diseases, especially in the absence of a specific drug treatment for NAFLD. As far as current drug development is concerned, small nucleic acid therapy is a major hotspot for future research and development.

### 3.5 Ketohexokinase

#### 3.5.1 Structure and function of KHK

Ketohexokinase (KHK) has two main protein isoforms, KHK‐A and KHK‐C.^236^ Among them, KHK‐C is selectively highly expressed in the liver, small intestine, and kidney, while KHK‐A is commonly (e.g., liver, kidney, intestine, etc.) expressed at a low level.^236^, ^237^ KHK‐C has a greater affinity for fructose and is the main phosphorylating enzyme involved in fructose metabolism.^236^, ^238^

Studies have shown that *KHK* LOF mutations cause primary fructosuria, allowing approximately 20% of the dietary fructose load to be eliminated in the urine.^239^ Interestingly, no increase in insulin and the presence of diabetic complications have been found in patients with this condition, so the disease is considered a clinically benign one.^240^, ^241^

Fructose is taken up into the cell from the intestine by glucose transporter type 5 and then rapidly and irreversibly phosphorylated to fructose‐1‐phosphate (F1P) catalyzed by KHK‐C.^242^, ^243^ This process causes a significant decrease in intracellular free phosphate and ATP (Figure 3D).^243^, ^244^, ^245^ Reduced phosphate levels lead to the activation of adenosine monophosphate deaminase, which converts adenosine monophosphate to inosine monophosphate, thereby driving the accumulation of purine products, including uric acid (UA), in the liver, intestine, and renal tubular cells. UA reduces AMPK activity, leading to reduced fatty acid oxidation. In addition, UA production may cause hyperuricemia,^246^ which increases the risk of gout, hypertension, metabolic syndrome, and cardiovascular disease, induce inflammation and oxidative stress, and may promote insulin resistance by impeding endothelial function.^247^ The buildup of UA crystals in the joints can lead to gout. In addition, F1P is cleaved into dihydroxyacetone phosphate (DHAP) and glyceraldehyde by aldolase B (ALDOB) after entering the cell,^248^ and after a series of metabolic transformations, it acts as a signaling molecule to promote adipose de novo synthesis and gluconeogenesis (Figure 3D),^245^, ^249^ leading to increased hepatic lipid synthesis and insulin resistance, and consequently, increasing the risk of developing obesity, NAFLD, and NASH. Studies have shown that the adverse metabolic effects of fructose intake may result in part from the activation of de novo liposynthesis (DNL), which is dependent on carbohydrate response element binding (ChREBP), providing a potential mechanistic basis for fructose‐induced metabolic dysfunction. Inhibition of KHK prevents hepatic ChREBP activation by fructose in vivo and thus protects against fructose‐induced DNL, steatosis, hypertriglyceridemia and hyperinsulinemia.^250^, ^251^

KHK expression is elevated in obese patients with NAFLD and in fructose‐fed mice, and it is hypothesized that it may cause NAFLD. Bennett et al.^252^ showed that hepatic KHK expression was positively correlated with NAFLD in mice through a phylogenetic study of inbred mice. Lanaspa et al.^243^ demonstrated that KHK is required for fructose‐mediated metabolic syndrome, TG formation, and hepatic steatosis in mice, and which endogenous fructose production and KHK activation within the kidney promotes the development of diabetic nephropathy and also reported for the first time that blockade of KHK may be an excellent treatment for the prevention and treatment of hereditary fructose intolerance. It has been shown that knockdown of intestinal KHK exacerbates fructose‐induced metabolic diseases, whereas specific knockdown or inhibition of hepatic KHK achieves beneficial effects opposite to those of intestinal,^244^ suggesting that the tissue specificity of KHK needs to be emphasized if it is to be used as a therapeutic target for metabolic diseases. Given the conflicting outcomes resulting from KHK deficiency in the liver and gut, new therapies for fructose‐driven NASH should focus on preventing inflammation, endoplasmic reticulum stress, and gut barrier deterioration, among others.^253^ In conclusion, these studies suggest that fructose metabolism in tissues expressing KHK‐c isoforms is critical for fructose‐induced metabolic diseases. Park et al.^254^ found that elevated KHK‐C mediates endoplasmic reticulum stress, leading to metabolic dysfunction. In addition, several studies have found that KHK overexpression may promote the proliferation of tumor cells such as breast cancer cells, hepatocellular carcinoma,^255^ pancreatic ductal adenocarcinoma,^256^ esophageal squamous cell carcinoma^257^ and glioma cells.^258^ This suggests that inhibition of KHK may offer new options for the treatment of several tumors.

Studies on *KHK* KO mice showed protection from fructose‐induced increases in body weight, fat mass, serum insulin, and fatty liver degeneration.^236^, ^243^ Softic et al.^259^ observed in mice with GalNAc‐siRNA targeting *KHK* mRNA that reduced KHK expression and improved hepatic steatosis and glucose tolerance without deleterious effects on renal function. This further suggests that KHK‐mediated fructose metabolism is critical for fructose‐induced metabolic diseases.

#### 3.5.2 Drug development of KHK

Genetic studies have shown that *KHK* LOF mutations are beneficial for fructose excretion, and inhibition of KHK can be used in the treatment of NAFLD/NASH by effectively suppressing fructose metabolism and its contribution to lipid accumulation, oxidative stress, inflammation, and insulin resistance. Drugs targeting KHK are currently in the pipeline, including the small molecule inhibitor PF‐06835919^260^ and the GalNAc‐siRNA drug ALN‐KHK (Table 5), which have demonstrated good efficacy (i.e., prevention of fructose‐induced hyperlipidemia, hyperinsulinemia, and steatosis).^261^

**TABLE 5:** Investigational drugs/technologies targeting KHK.

Drug name	Originator company	Status	Target disease	References
PF‐06835919	Pfizer Inc	Phase 2 clinical	Metabolic abnormalities; NAFLD	^261^, ^262^
ALN‐KHK	Alnylam Pharmaceuticals Inc	Phase 2 clinical	Diabetes mellitus; T2D	^261^, ^263^
LY‐3478045	Eli Lilly & Co	Phase 1 clinical	NASH; Diabetes mellitus	^264^
LY‐3522348	Eli Lilly & Co	Phase 1 clinical	Fibrosis; Liver disease; NASH	^265^

In conclusion, KHK is one of the rate‐limiting enzymes of fructose metabolism and has great potential in various aspects of fructose‐mediated regulation of hepatic lipogenesis and insulin resistance. Therefore, inhibition of KHK has therapeutic implications for NAFLD, NASH, T2D, and other fructose‐mediated metabolic diseases. In addition, it is also expected to enhance a new modality for the treatment of several tumors. Although there are not many drugs targeting KHK at the moment, it is believed that more drugs will meet you in the future as more attention is paid to it. Especially with the development of delivery technology, the use of nucleic acids to inhibit KHK becomes a trend in the future.

### 3.6 Cell death‐inducing DFF45‐like effector B

#### 3.6.1 Structure and function of CIDEB

Cell death‐inducing DFF45‐like effector B (*CIDEB*) is located on human chromosome 14q11 and is highly expressed in human liver cells.^213^, ^266^ In addition, it is lowly expressed in renal, small intestinal, and pancreatic β‐cells.^267^ It has been shown that CIDEB is a paramount lipid droplet (LD) surface protein, mainly localized on LD and smooth endoplasmic reticulum,^268^ which regulates lipid metabolism and LD fusion and growth by binding to apo B to promote VLDL lipidation and maturation.^213^, ^267^ Studies in *CIDEB* ^−/−^ mice have also confirmed that CIDEB plays a multifunctional role in controlling hepatic lipid secretion, storage, and synthesis, preventing diet‐induced obesity, insulin resistance, hepatic steatosis, and inflammation.^267^, ^269^ Among other things, it is believed that resistance to high‐fat diet‐induced obesity in defective mice is achieved through downregulation of fatty acid synthesis by SREBP1c.^270^ In addition, knockdown of *CIDEB* in mice has been shown to significantly reduce the biogenesis of VLDL transporter vesicles. Ping et al.^266^ reported for the first time the correlation between *CIDEB* gene promoter methylation levels and overweight or obesity in adult abdominal adipose tissue. Thus, the *CIDEB* gene plays an important role in maintaining lipid homeostasis and energy metabolism throughout the body.

In 2022, the WES of more than 540,000 people found that people who carried the *CIDEB* LOF mutation were about 54% less likely to develop nonalcoholic cirrhosis,^213^ about 53% less likely to develop NASH,^213^ and about 49% less likely to have a risk of liver cancer compared with noncarriers.^213^ There were also improvements in T2D and obesity.^213^

Based on these studies, the researchers investigated the mechanism of the CIDEB mutation by siRNA silencing of the *CIDEB* gene in a human hepatocellular carcinoma cell line to mimic LOF and found that the mutation prevented fat from accumulating in the hepatocytes and produced smaller LD.^213^ This suggests that the disease‐preventive effect caused by *CIDEB* LOF may be related to a reduction in the accumulation and fusion of LD in fatty liver cells. In conclusion, CIDEB plays a crucial role in maintaining the balance of lipid levels in the liver by controlling various processes such as the merging of LD, the production of lipids, and the release of lipids in response to changes in metabolic activity. Its regulatory function is essential for overall hepatic lipid homeostasis.^270^ Therefore, therapies that effectively mimic these beneficial mutations by blocking CIDEB expression or function may help prevent or treat NASH and related diseases.

#### 3.6.2 Drug development of CIDEB

Based on the above studies, it has been demonstrated that CIDEB plays an important role in maintaining systemic lipid homeostasis and energy metabolism. It has also been shown that therapies that effectively mimic these beneficial mutations by blocking CIDEB expression may help prevent or treat NASH and related diseases. However, it has been suggested that the role of CIDEB in the gut is different from that of the liver, and that CIDEB deficiency prevents lipid efflux from enterocytes, leading to excessive accumulation of lipids in the intestinal mucosa.^271^ Therefore, targeting CIDEB to treat disease also requires consideration of its tissue specificity.

Due to the relatively late discovery of the role of CIDEB in diseases such as NAFLD in humans, no related drugs have yet entered clinical trials. However, Regeneron has partnered with Alnylam to develop a siRNA therapeutic candidate that silences the *CIDEB* gene, intended for the potential treatment of NASH and cirrhosis.^213^ Additionally, The Board of Regents of the University of Texas System, United States, Regeneron Pharmaceuticals, Inc. and other organizations or companies have similarly conducted research on siRNA drugs targeting CIDEB, suggesting that CIDEB is of interest as a NASH‐associated disease target has attracted much attention.^272^, ^273^ There are currently no drugs on the market for NAFLD, which suggests a broader market for CIDEB inhibitors. Of course, to develop CIDEB inhibitors, further validation of their safety after inactivation is needed. In conclusion, CIDEB has a broad research and development prospect as a potential new target for the treatment of NAFLD/NASH.

### 3.7 G protein‐coupled receptor 75

#### 3.7.1 Structure and function of GPR75

The *CPR75* gene is located on human chromosome 2p16 and encodes a novel protein of 540 amino acids in length.^274^ GPR75 presents the characteristic structural features of GPCRs, namely seven transmembrane structural domains, an N‐terminal N‐glycosylation site, and numerous serine and threonine phosphorylation sites at the C‐terminus.^275^ However, it is unique from other GPCRs in that it contains approximately 200 amino acids, a 92‐residue putative intracellular third loop, and an extra‐long C‐terminal tail of 169 residues.^276^ GPR75 is primarily expressed in the brain and central nervous system.^275^ In addition, it is expressed at low levels in alternate tissues including the heart and kidney.^274^

In 2021, the WES association study of more than 640,000 humans with body mass index (BMI) identified the rare LOF in *GPR75* as being associated with obesity prevention.^277^ Compared with noncarriers, *GPR75* LOF heterozygous carriers have a lower BMI, an approximately 54% lower risk of obesity, and a reduced chance of developing T2D.^277^ In a high‐fat diet‐induced obese mouse model, KO mouse *GPR75* resisted weight gain and improved blood glucose as evidenced by lower insulin levels and increased insulin sensitivity, especially in *GPR75* ^−/−^ mice.^277^, ^278^, ^279^ This suggests that GPR75 inhibition may provide novel therapeutic approaches for T2D and other diseases associated with insulin hypersecretion and resistance.^279^ Choi et al.^280^ similarly found that a missense variant of GPR75 (p.T27A) showed a similar direction of action as the BMI‐associated protein truncation variant. According to statistics, about 3 billion people worldwide are currently overweight or suffering from obesity, which shows that there is a broad market for weight loss drugs. Given the above findings and research, GPR75 is expected to be a novel potential target for the treatment of obesity.

In addition, Garcia et al.^274^ suggested that GPR75 could be a target for cardiovascular disease. GPR75 is the first specific receptor for 20‐hydroxyeicosatetraenoic acid (20‐HETE), and studies have shown that 20‐HETE signals through GPR75 to influence vascular function.^281^, ^282^ Reduction of 20‐HETE‐mediated hypertension, vascular dysfunction and vascular remodeling in the *GPR75* KO animal model.^274^, ^275^ It suggests that knockdown of GPR75 prevents 20‐HETE‐mediated vascular remodeling and hypertension. In addition, GPR75 plays an important role in the pathogenesis of pulmonary hypertension and may attenuate cAMP‐dependent signaling while enhancing lung contraction in response to hypoxia.^283^, ^284^ Deletion of the GPR75 prevents hypoxia‐induced pulmonary vasoconstriction and hypertension.^283^ GPR75 receptors are involved in the activation of intracellular signaling known to be stimulated in malignant transformation of cells, leading to a more aggressive phenotype of prostate cancer cells.^281^, ^285^ Targeting the 20‐HETE/GPR75 pathway is expected to interfere with the malignant progression of prostate tumor cells.

In conclusion, GPR75 is a potentially biologically and pharmacologically significant receptor that plays a crucial role in many diseases such as obesity, cancer and metabolic syndrome. Inhibition of GPR75's may have beneficial effects on diverse diseases including cerebrovascular disease, cardiovascular disease, diabetes, cancer, and obesity.^275^, ^286^

#### 3.7.2 Drug development of GPR75

GPR75 was considered an orphan receptor some years ago and has only begun to be deorphanized in recent years.^287^ However, GPR75 is still poorly studied. Although GPR75 lowering was found to be beneficial in resisting weight gain,^275^, ^277^ it has been shown to have neuroprotective effects through CCL5 activation.^276^, ^288^ Therefore, GPR75 still needs more research to discover its physiological functions. This also explains the relatively small number of drugs targeting GPR75.

Fortunately, Regeneron has now partnered with AstraZeneca to develop small molecule therapies for GPR75,^275^ aimed at treating obesity and related complications. In addition, BeBetter Med's oral small molecule GPR75 inhibitor, BEBT‐809, is in IND regulated trials.^289^ Alnylam Pharmaceuticals, Inc. has conducted research on siRNA drugs targeting *GPR75* mRNA for the prevention and treatment of weight disorders, such as obesity and related diseases.^290^ Regeneron Pharmaceuticals, Inc. is targeting GPR75 to develop a method to inhibit its expression using ASO or the CRIPSR/Cas system in hopes of treating obesity.^291^ It is believed that with the development of gene therapy, GPR75 will be even more promising to play a full role in the treatment of diseases. With respect to the available findings, GPR75 is a prominent player in the control of metabolism and glucose homeostasis, and may also be a novel therapeutic target to combat metabolic disorders caused by obesity.

### 3.8 Inhibin βE

#### 3.8.1 Structure and function of INHBE

Inhibin βE (*INHBE*) gene is mainly expressed in the liver and encodes the INHBE subunit. The INHBE subunit is a protoprotein consisting of a prepeptide structural domain and a mature structural domain.^292^ The prepeptide structural domain is cleaved after protein synthesis to form mature activin E, whose expression is regulated by the fatty acid sensor PPAR‐α.^293^, ^294^ INHBE is a growth factor involved in the regulation of hepatocyte growth and differentiation.^295^

Analysis of WES data from more than 360,000 individuals of European ancestry revealed that the *INHBE* LOF mutation contributes to a reduction in the prevalence of abdominal obesity, metabolic syndrome, coronary heart disease, and T2D.^292^ Akbari et al.^296^ found that mutations in the *INHBE* gene favored fat distribution and the prevention of metabolic diseases such as diabetes by sequencing the exomes of more than 610,000 individuals from three population‐based cohorts in the United Kingdom, Sweden, and Mexico, which is consistent with the results of Deaton et al.^292^ Sugiyama et al.^295^ found that hepatic expression of *INHBE* mRNA was upregulated in insulin resistant and obese humans. It is also suggested that INHBE causes muscle‐reducing obesity by decreasing fat utilization and can alter whole‐body energy metabolism under conditions of insulin resistance in obesity.^295^ In addition, a study reported the association between increased INHBE expression and insulin resistance and obesity in humans and mice, confirming that siRNA selectively silences the *INHBE* gene, thereby suppressing the increase in body weight and fat mass.^295^ Griffin et al.^293^ suggested that mice deficient in INHBE develop insulin resistance likely because of activin E on adipose tissue lipolysis. Adam et al.^294^ had the same insight. They also suggested that elevated INHBE leads to adipose dysfunction, which may be particularly relevant to patients with NASH and T2D.^294^ In addition, Cao et al.^297^ and Wen et al.^298^ similarly found that INHBE may be associated with NAFLD. Further studies of rare LOF variants in INHBE did not reveal any associated adverse effects, suggesting that INHBE may be a promising new target for the treatment of lipid metabolism, diabetes, and cardiovascular disease.^292^ Besides, Xu et al.^299^ suggested that INHBE may be involved in the pathogenesis of clear cell renal cell carcinoma and function as a tumor promoter.

Currently, INHBE has not been studied enough, and there are few insights into the mechanism of its function, and more research is still needed to discover its physiological function.

#### 3.8.2 Drug development of INHBE

The above studies suggest that inhibition of INHBE may promote healthy obesity and improve glycemic control. The *INHBE* gene is expressed in the liver,^292^ which is more favorable for the development of RNAi drugs using the GalNAc delivery platform. In addition, drugs targeting INHBE may have different biological mechanisms than those currently used to treat CHD and T2D, and are expected to complement current therapeutic approaches.

Due to the lack of research on INHBE, there are currently no drugs targeting INHBE in the clinic. Fortunately, Alnylam has utilized its liver KARIA platform for nucleic acid drug development targeting INHBE.^300^, ^301^, ^302^ Moreover, Regeneron Pharmaceuticals, Inc. has initiated a research layout of INHBE inhibitors designed to prevent or treat metabolic disorders and/or cardiovascular disease.^303^

### 3.9 Others

In fact, there are many more LOF targets identified through genetic approaches such as GWAS, WES, and WGS that produce favorable clinical effects. They are involved in several disease areas, such as cardiovascular diseases, oncology, and central nervous system diseases.^304^ Among them, WGS for rare diseases is well established. In addition, WGS‐based analyses have revealed multiple cancer‐driving events located in noncoding regions of DNA, such as promoters, enhancers or miRNA‐encoding genes.^61^ A few examples of relevant targets are given below. In a WES of smoking phenotypes in up to 749,459 individuals, Rajagopal et al.^305^ found that LOF mutations in *CHRNB2*, encoding the β2 subunit of the α4β2 nicotinic acetylcholine receptor, were associated with a 35% lower risk of heavy smoking. Darrah et al.^306^ found by GWAS that *AGTR2* deletion or antagonism prevented pulmonary cystic fibrosis and is expected to be a novel therapeutic target for pulmonary cystic fibrosis. Several studies have shown that *APOC3* LOF mutations reduce TC levels, risk of ischemic cardiovascular disease, and risk of coronary heart disease.^307^, ^308^ Salem et al.^309^ found by GWAS that *COL4A3 rs55703767*, *DDR1 rs118124843*, and *COLEC11 rs12615970* prevented diabetic nephropathy. Emdin et al.^310^ performed an association analysis of 3759 LOF variants with metabolic traits, cardiometabolic diseases, and other disorders in 405,569 UK Biobank participants and found that the *GPR151* LOF variant prevented obesity and T2D, the *IL33* LOF and *GSDMB* LOF variants prevented asthma and allergic diseases, the *IFIH1* LOF variant prevented hypothyroidism, and the *PDE3B* LOF variant was associated with increased height, improved body fat distribution, and prevention of hypercholesterolemia and CAD. The association between these LOF variants and disease prevention provides a theoretical basis for ameliorating disease through the development of inhibitors of the relevant targets. The identification of beneficial LOF variants also contributes to the development of disease therapeutics.

## 4 CONCLUSIONS AND FUTURE PROSPECTS

Discovering and developing new drugs is a difficult and high‐risk process. There is growing evidence that targets with supporting genetic data are a positive indicator of good clinical trial results for their drugs.^9^ The potential for drug discovery using human genetic approaches is enormous, and the development of new drugs based on their validated targets has become an emerging trend in new drug development, especially when favorable results from LOFs are used for drug development. Utilizing beneficial LOF mutations as targets in drug development has potential as it can help develop more precise and effective drug regimens. For example, drugs can be developed in a targeted manner to treat diseases associated with these mutations, thereby reducing the adverse effects on normal cells and improving the safety and tolerability of the drugs; by understanding a patient's genomic information, a personalized drug treatment plan can be developed for the patient based on the characteristics of his or her LOF mutations, improving the precision and effectiveness of the treatment.

WES, WGS, and GWAS provide important support for precision medicine, afford new insights into drug development for some under‐recognized candidate genes, and help identify safety, alternative indications, and repurposing opportunities for potential targets. This review confirms the importance of human genetics in the development of new drugs by focusing on the biological functions, targeted drugs, and future research perspectives of eight targets (PCSK9, ANGPTL3, ASGR1, HSD17B13, KHK, CIDEB, GPR75, and INHBE), in which LOF mutations lead to a reduced risk of disease. In particular, lipid‐lowering therapies approved in recent years, such as PCSK9 and ANGPTL3 inhibitors, derive from LOF mutations that have been validated in a small number of human individuals, further confirming the importance of human genetics in new drugs development. Furthermore, a brief overview of the associations between other beneficial LOF mutations and disease complements the druggable targets. Of course, the exact effect will need to be determined based on the specifics of the gene and mutation. It is important to note that LOF mutations may also lead to adverse effects in other areas, and thus the potential benefits and risks need to be considered together.

While large‐scale data have made it easier to identify rare genetic variants that are important for health and disease, as these databases become larger and include other genomic data beyond the genome, including transcriptomics, proteomics, and even metabolomics data, their analysis becomes a major challenge, which will create multiple challenges for bringing potential drug targets to the therapeutic level. For example, most GWAS findings are located in noncoding regions, making genetic mechanisms of action more difficult to elucidate; WES requires very large sample sizes and a large number of cases; Intervention in the pathway may have adverse effects or will not provide sufficient clinical benefit for the target disease; In most existing studies, subjects of European origin are elevated, and there is a lack of participants from diverse populations. Genomic research represents a major ethical and scientific challenge that may, among other things, exacerbate existing inequalities in health care.^51^ Fortunately, developments in artificial intelligence and machine learning tools are bringing light to data analysis,^54^, ^311^, ^312^ and their application is not limited to predicting biological targets of existing drugs or compounds but also to identifying new therapeutic targets for any disease of interest, promising to revolutionize drug development and overcome barriers in the drug discovery pipeline.^312^, ^313^ In addition, CRISPR‐based high‐throughput screening can rapidly validate GWAS results to identify genetic factors of disease, thereby identifying new drug targets and drivers of resistance.^50^, ^314^

In conclusion, new methods and techniques for drug target discovery provide emergent ideas and tools for drug development. Genetics‐based target discovery methods enable researchers to discover potential drug targets more efficiently, rendering new opportunities for the development of new drugs. With both opportunities and challenges, identifying drug targets from human‐specific disease pathways and drug development will be a hot topic in the future. In addition, further research in genetics on targets and the integration of genetics into the clinical drug development process will be more conducive to promoting the success and approval rates of drug development and expanding the range of indications for drugs that have been approved or are in clinical trials. In summary, target‐based drug discovery remains the key to drug development, but the future direction of R&D needs to incorporate more technologies and approaches. It requires us to turn attention to more comprehensive and predictive issues, and incorporate modern technologies to look at diseases holistically in order to meet the needs of public health, respond to the challenges of the pharmaceutical industry, and make a more significant contributions to medical research and drug development, thereby further accelerating the development of healthcare.

## AUTHOR CONTRIBUTIONS

Xiaoxia Zhang generated the main idea, performed literature search and analysis, and discussed and aligned all literature data and wrote the manuscript and prepared figures and tables. Yuanlei Fu, Haiqiang Cao, and Aiping Wang conceived the manuscript. Wenjun Yu and Yan Li discussed and reviewed the manuscript. All authors reviewed and approved the final manuscript.

## CONFLICT OF INTEREST STATEMENT

The authors declare that they have no conflict of interest.

## Data Availability

All data generated or analyzed during this work are included in this published review.
